# Chronic Kidney Disease Is Associated With Increased All-Cause Mortality Risk Among Older Black Adults

**DOI:** 10.1093/geroni/igae064

**Published:** 2024-06-28

**Authors:** Ryon J Cobb, Roland J Thorpe, Keith N Norris

**Affiliations:** School of Social Work, Rutgers University, New Brunswick, New Jersey, USA; Department of Health, Behavior, and Society, Johns Hopkins Bloomsburg School of Public Health, Baltimore, Maryland, USA; Division of General Internal Medicine and Health Services Research, University of California, Los Angeles, California, USA

**Keywords:** Biomarkers, Minority health, Renal disease

## Abstract

**Background and Objectives:**

Older patients diagnosed with chronic kidney disease (CKD) have a higher risk of all-cause mortality than the general population. However, there is limited information available on how CKD relates to all-cause mortality among Black adults in the United States. We aimed to investigate how CKD relates to all-cause mortality risk among older Black adults.

**Research Design and Methods:**

This study draws on a subsample of self-identified Black participants (*N* = 1 393) from the Health and Retirement Study ages 52 to 96 who completed the anthropomorphic and biomarker supplement in 2006/2008. Our measure of CKD derives from serum cystatin C-based using dried blood spots, and all-cause mortality derives from the National Death Index and a key informant within the household from 2006 to 2019.

**Results:**

Twenty-nine percent of respondents died during the study period, whereas 31% had CKD. The mean age of the entire sample is 64.52. Results from our Cox proportional hazards models showed that CKD was independently associated with an increased risk of death from all causes among older Black participants in a model that adjusted for demographics, behavioral, clinical, and health characteristics.

**Discussion and Implications:**

Results from our study confirm that CKD is associated with increased risk of death from all causes among older Black adults. Future studies should examine whether changes in CKD over time relate to all-cause mortality risk among older Black adults.

**Translational Significance:** This study demonstrates that chronic kidney disease (CKD) is a risk factor for all-cause mortality among older Black adults from a nationally representative survey of adults aged 50 and older in the United States. The findings from this study underscore the need for community-based organizations that work with high-risk populations to educate older Black adults about their risks for CKD and take steps to speak with their healthcare providers to receive screening for CKD.

Despite numerous policy initiatives, chronic kidney disease (CKD) remains a national public health problem among adults in the United States. Findings from the United States Renal Data System (USRDS) suggest that the overall prevalence of CKD among U.S. adults, defined as estimated glomerular filtration rate (eGFR) < 60 mL/min per 1.73 m^2^ (1), is 14.0% in the general population and 27% among adults aged 50 and older (2). Research suggests that doctor-diagnosed CKD remains a leading source of death among adults aged 50 and older (3). Yet, the United States Preventative Services Task Force does not have a screening recommendation for this “silent killer” in the United States, as 90% of adults with CKD do not exhibit symptoms until the late stages of the disease, when older adults may need dialysis or a kidney transplant (4). Information from the USRDS indicates that the total Medicare Fee-for-Service spending for individuals with a CKD diagnosis was $86.1B in 2021, representing nearly a quarter of total Medicare Fee-for-Service expenditures (2).

The present study examines how CKD relates to all-cause mortality risk among older Black participants from a nationally representative survey of older adults in the United States. Though this study is not the first to investigate the links between CKD and mortality risk (5), fundamental differences exist between the current study and prior research on this topic. First, though research clearly shows that CKD is a factor that predicts grip strength, mobility limitation, cognitive decline, cardiovascular disease, and end-stage renal disease (6–8), mortality is rarely an endpoint considered in research on the health-related consequences of CKD among older adults. Studies on the relationship between CKD and mortality risk also tend to rely on data from hospital patients with a doctor’s diagnosis of CKD (9). Though important, an estimated 9 out of 10 adults with CKD are unaware they have this condition, which, in turn, limits our understanding of the link between CKD and mortality risk among adults (2). Here, we use data from the Health and Retirement Study (HRS) to overcome this limitation. This panel study surveys a representative sample of adults aged 50 and older in the United States, allowing us to examine the relationship between CKD and mortality risk, particularly at pre-end-stage renal disease (ESRD) stages.

Additionally, most studies on CKD as a risk factor for poorer health tend to rely on creatinine-based measures of kidney function among older adults. In contrast, the HRS contains a Cystatin C-based measure of kidney function. Unlike creatinine-based measures of kidney functioning, Cystatin-C-based measures of kidney functioning are not influenced by diet and muscle mass, and many scholars of older populations contend that it is a better predictor of kidney function than creatinine-based measures (10).

Second, findings from HRS data suggest that kidney dysfunction (11) and CKD are more common among older Black adults relative to their Hispanic and White counterparts. Yet, research on the relationship between racial identification, CKD, and mortality risk tends to adopt an across-racial group framework that focuses on CKD as a moderator of the relationship between racial identification and mortality in the general population (12,13). This framework helps advance research on CKD and mortality risk by adopting a within-racial groups approach to spotlight how CKD predicts all-cause mortality risk among older Black adults. Based on prior research, we hypothesized that CKD would be associated with all-cause mortality risk among older Black participants, even after adjusting for demographic, clinical, and behavioral factors.

The possible mechanisms in which CKD contributes to increased mortality risk from death from all causes may include several components. Tonelli, writing CKD and mortality, argues that current guidelines identify individuals with CKD as being at high risk for cardiometabolic conditions such as hypertension, obesity, diabetes, high cholesterol, and inflammation (5). Therefore, cardiometabolic risk factors may explain some of the hypothesized associations between CKD and all-cause mortality risk among older adults (14,15). Research has long uncovered relationships between health behaviors (e.g., alcohol consumption, tobacco, and medication usage) and CKD, so behavioral risk factors may explain some of the hypothesized relationships between CKD and all-cause mortality risk. Norris, writing on socioeconomic disparities related to CKD and diabetes, contends that education and income serve as barriers to preventing and slowing the progress of CKD on health among adults (16). Therefore, socioeconomics may explain some of the hypothesized relationships between CKD and all-cause mortality risk.

This article proceeds as follows. First, we describe our data in detail and review the methods we use to examine how CKD relates to all-cause mortality risk among older Black adults. Following this, we show our analytic results showing how and why CKD relates to all-cause mortality risk among older Black adults. We conclude this study by summarizing our findings and implications for future research on the social determinants of health among older Black adults in the United States.

## Data

Participants were drawn from the HRS, a nationally representative panel study of adults 50± years of age and their spouses. It was designed to monitor age-related changes in health and labor force transitions among older adults in the United States. The HRS is conducted by the University of Michigan and funded by the National Institute of Aging (grant no. National Institute on Aging U01AG009740). In 2006, trained interviewees collected blood, body measurements, and physical examinations from half of the respondents during the in-person interviews. Interviewers collected the same information from the other half of the 2006 sample in 2008. We pooled the two assessments, and all self-identified Black respondents with relevant data were included in the analysis (i.e., biomarkers, demographic covariates, and vital status) (17).

### Measures

#### All-cause mortality

Mortality information was based on the National Death Index (through 2011) and personal communication between HRS staff and respondents’ family members (through 2019). Survival time was computed from the day of the 2006/2008 biomarker assessment until death for participants who died. We treat respondents who survived until follow-up as censored, and their waiting time ended in the month of their last interview.

#### Chronic kidney disease

Our outcome, CKD, was developed using the biomarker Cystatin C. Cystatin C is a protein produced by all nucleated cells, filtered by the glomerulus, and considered a reliable marker of kidney function. During the in-home assessment, HRS interviewers collected dried blood spots and assayed Cystatin C by pricking participants’ fingers with a sterile lancet and depositing the blood droplets onto specially treated filter paper (17). The HRS applied the natural logarithm to normalize the skewed distribution of Cystatin C. Other eGFR constructions that create CKD rely on converting creatinine or Cystatin C (1). The HRS’s 2010/2012 biomarker assessment does not include a measure of creatinine. Therefore, following the Chronic Kidney Disease Epidemiology Collaboration (CKD-EPI), we used the 2012 CKD-EPI Cystatin C equation to measure eGFR based on Cystatin C, age, and gender. Following prior research, we defined CKD based on an estimated glomerular filtration rate of less than 60 mL/min per 1.73 m^2^ (18).

#### Cardiometabolic risk

Seven biomarkers were used to measure cardiometabolic risk: glycosylated hemoglobin (HbA1c), C-reactive protein (CRP), high-density lipoprotein (HDL) cholesterol, total cholesterol (TC), waist circumference, resting heart rate, and blood pressure. HRS interviewers used an Omron HEM-780 N Monitor to collect information on systolic and diastolic blood pressure and resting heart rate. Blood pressure and resting heart rate were collected simultaneously as respondents sat down with both feet on the floor using an automated blood pressure monitor placed on the respondent’s left arm, with approximately 45–60 seconds between each measurement, and the difference between systolic and diastolic blood pressure was calculated to measure pulse pressure. Waist circumference was measured by wrapping tape around the respondent’s waist as they stood and raised their arms. The HRS interviewer collected each measure three times, and we used the average of non-missing measures for this study. The HRS biomarker values for HbA1c, CRP, HDL, and TC were assayed from dried blood spots. Additional details are provided regarding the protocol, special instructions, and other criteria required to measure each indicator correctly (17). We created a cardiometabolic risk count variable by summing the biomarkers where the respondent scored in the top 25th percentile (except HDL cholesterol, where risk is defined as the lowest 25%), indicating a high risk.

#### Other covariates

Demographic covariates included chronological age (at baseline interview) and gender (1 = men, 0 = women). We also included indicators of blood pressure and cholesterol medication usage; we used 2 measures of health behaviors: the number of days per week the respondent consumes alcoholic beverages and smoking status (i.e., current smoker, former smoker, and nonsmoker). Socioeconomic characteristics include years of completed education and logged yearly household income.

### Statistical Analysis

Sample characteristics (e.g., mean, standard deviation, and proportion) were summarized for our analytic sample and by CKD status in Table 1. Given that the proportional hazards assumption was met, we used Cox proportional hazards regression analysis to test whether CKD was associated with all-cause mortality risk among older Black participants in Table 2. Our study implements the following progressive modeling strategy. Model 1 controlled for age and gender to show the relationship between CKD and all-cause mortality risk after adjusting for essential demographic characteristics. Model 2 controlled for these demographic characteristics listed in Model 1 and health characteristics to test whether the association between CKD and all-cause mortality risk is independent of cardiometabolic risk. Model 3 included the covariates from Model 3 as well as medication usage. Model 4 controlled for the characteristics in Model 3 and health behaviors (i.e., smoking status and alcohol consumption) to test whether the relationship between CKD and all-cause mortality risk is net of health behaviors that also increase the risk of death from all causes. Our final model (Model 5) included the covariates from Model 4 to examine whether the relationship between CKD and all-cause mortality risk is independent of socioeconomics. Sampling weights are applied to account for the complex sample design of the HRS. All analyses were run using STATA 16, and we presented the hazard ratios (HR) and 95% confidence intervals (CI) for the models in Table 2.

**Table 1.** Descriptive Statistics, 2006/2008 Health and Retirement Study (*N* = 1 393)

Variables	Full Sample *N* = 1 393					CKD *n* = 456			No CKD *n* = 937		
	%	Mean	*SD*	Min	Max	%	Mean	*SD*	%	Mean	*SD*
Vital status											
Deceased	29					39			19		
Alive	71					61			81		
Mean survival time for descendants		76.69	10.21	57.5	102.8		78.96	10.67		78.96	10.67
CKD											
No	69										
Yes	31										
*Demographic characteristics*											
Gender											
Men	42					45			40		
Women	58					55			60		
Chronological age, years		64.52	9.50	52	96		7.02	11.31		62.31	7.63
*Health risks*											
Cardiometabolic risk		1.71	1.13	0	5		1.78	1.23		1.68	1.09
Medication usage											
Cholesterol medication											
No	81					75			84		
Yes	19					25			16		
Blood pressure medication											
No	36					28			40		
Yes	64					72			60		
*Health behaviors*											
Smoking status											
Never smoked	42					34			44		
Former smoker	38					42			36		
Persistent smoker	20					24			18		
Number of drinks per week		0.59	1.32	0	7		0.49	1.29		0.63	1.33
*Socioeconomic status*											
Years of education		11.91	3.26	0	17		11.87	3.55		12.33	3.04
Yearly household income (logged)		9.99	1.55	0	14.27		9.61	1.29		10.14	1.62

*Notes*: CKD = chronic kidney disease; Max = maximum; Min = minimum; *SD* = standard deviation.

**Table 2.** Mortality Risk Associated With CKD and All-Cause Mortality Risk Among Older Blacks, 2006/2008 Health and Retirement Study (*N* = 1 393)

Variable	Model 1	Model 2	Model 3	Model 4	Model 5
	HR (95% CIs)	HR (95% CIs)	HR (95% CIs)	HR (95% CIs)	HR (95% CIs)
CKD	2.24 (1.73–2.90)	2.23 (1.72–2.89)	2.18 (1.67–2.85)	2.14 (1.64–2.80)	2.08 (1.59–2.72)
Demographic characteristics					
Chronological age, years	0.69 (0.55–0.87)	0.69 (0.55–0.88)	0.69 (0.55–0.87)	0.72 (0.57–0.91)	0.70 (0.55–0.88)
Female	1.00 (0.97–1.04)	1.01 (0.97–1.04)	1.01 (0.97–1.04)	1.01 (0.98–1.04)	1.01 (0.97–1.04)
Health risks					
Cardiometabolic risk		1.06 (0.94–1.19)	1.06 (0.94–1.19)	1.05 (0.93–1.17)	1.04 (0.93–1.16)
Cholesterol mediation			1.08 (0.82–1.41)	1.07 (0.81–1.40)	1.08 (0.83–1.42)
Blood pressure medication			1.09 (0.83–1.42)	1.14 (0.87–1.50)	1.14 (0.86–1.49)
Health behaviors					
Former smoker				1.25 (0.97–1.62)	1.26 (0.97–1.63)
Current smoker				2.38 (1.71–3.30)	2.26 (1.62–3.16)
Number of drinks per day				0.96 (0.86–1.07)	0.96 (0.87–1.07)
Socioeconomic status					
Years of education					0.98 (0.95–1.02)
Yearly household income (logged)					0.92 (0.87–0.98)

*Notes*: CI = confidence interval; CKD = chronic kidney disease; HR = hazard ratio.

## Findings

Twenty-nine percent of respondents died during the study period. The mean survival time for decedents was 76.69 months (standard deviation [*SD*] 10.21 months; range 57.5–102.8 months), whereas 31% of our sample had CKD. Nearly 60% (58%) of our sample comprises women. On average, respondents were 64.2 years of age (*SD* = 9.5). The mean cardiometabolic risk score was 2.0 (*SD =* 1.29). Nearly 20% of respondents reported taking cholesterol medication, and 64% were on blood pressure medication. Forty-two percent of respondents in our sample reported never smoking, 38% were former smokers, and 20% were persistent smokers. On average, respondents in our sample consumed 0.59 (*SD* = 1.32) alcoholic beverages weekly. Respondents in our sample had an average of 11.9 (*SD =* 3.26) years of education and a (logged) household yearly income of 9.99 thousand dollars (*SD* = 1.15).

The association between CKD and all-cause mortality risk among older Black adults is shown in Table 2. Controlling for the demographic characteristics, Model 1 reveals those respondents with CKD (HR: 2.24, 95% confidence interval [95% CI]: 1.73–2.90) had a higher risk of death from all causes relative to those who did not have CKD in demographic-adjusted models. In Model 2, the relationship between CKD and all-cause mortality risk remained significant after adjusting for cardiometabolic risk (HR: 2.23, 95% CI: 1.72–2.89). Model 3 includes adjustments for cholesterol and blood pressure medication usage, and we find that older adults with CKD had a higher risk of all-cause mortality than older Black adults who did not have CKD (HR: 2.18, 95% CI: 1.67–2.85). This relationship was held after further adjustments for health behaviors (HR: 2.14, 95% CI: 1.64–2.80) and economic status (HR: 2.08, 95% CI: 1.59–2.72).

## Discussion

The present study extends present knowledge on the consequences of CKD among adults by drawing on a subsample of older Black participants from a nationally representative study to examine how CKD relates to all-cause mortality risk among older Black adults. Our primary goal was to investigate how CKD relates to all-cause mortality risk among older Black adults in the United States. Our secondary goal in this study was to examine whether the association between CKD and all-cause mortality risk was independent of clinical (e.g., cardiometabolic risk), behavioral factors (e.g., tobacco usage, alcohol consumption, and medication usage), and economic factors. Consistent with our hypothesis, findings from this study suggest that CKD is associated with a higher risk of death from all causes among older Black adults in the United States, and while attenuated, remained significant after further adjustments for clinical, behavioral, and economic covariates.

The positive association between CKD and all-cause mortality risk observed in a subsample of older Black adults from a nationally representative survey of adults above the age of 50 is consistent with findings from research on non–dialysis-dependent CKD and death from all causes. Although a number of these studies focused on patients with doctor-diagnosed CKD exclusively (9), this study is the first to our knowledge to examine how CKD relates to all-cause mortality risk among older Black adults from a nationally representative study. As noted earlier, the respondents in our study are adults in the pre-ESRD phase. Hence, our findings provide additional support for the notion that diagnosed and undiagnosed CKD is a salient risk factor for all-cause mortality risk among older Black adults.

Our findings from this investigation suggest that clinical, behavioral, and economic factors do little to explain the relationship between CKD and all-mortality risk among older Black adults. For instance, Afkaria et al. (19) found that a creatinine-based measure of CKD was associated with greater mortality risk among a subsample of Black adults from a nationally representative study of adults in the United States across ten years. A separate study found that CKD was independently associated with all-cause mortality among respondents in a survey that examined genetic and environmental risk factors associated with cardiovascular disease in Black individuals in Jackson, Mississippi (20). This study is the first to investigate the link between CKD and all-cause mortality risk among older Black adults from a nationally representative survey.

Future research should examine how social adversity experienced in early life relates to the development of CKD among older Black adults (21). As noted earlier, older Black adults are at greater risk of CKD and ESRD than their White counterparts. These disparities are independent of economics, nativity status, lower access to care, or other cardiometabolic risk factors (22). Some scholars believe advanced age-associated decline in mortality among older Black adults may lead to selective survivorship and contribute to racial disparities in ESRD. However, it remains unclear how early life adversity translates into differences in age-associated decline in kidney function. One perspective, the biological imprint framework, contends that early life adversities permanently alter one’s organ structure. In contrast, the pathway framework suggests that early life adversity triggers lifelong trajectories of health-related advantages or disadvantages that increase the rate of renal aging. However, we are unaware of any research that explicitly examines how and why early life adversity contributes to renal aging among older Black adults.

Decades of National Institutes of Health-funded investigations have uncovered an underlying genetic basis for renal aging. Polygenic risk scores (PRS) for kidney function, which estimate a person’s genetic liability to a renal trait or disease, are one example. Research on PRS for kidney function quickly transitions from estimation to determining when and how clinicians will adopt these scores into clinical settings. Though significant, PRS for kidney function only estimates the relative risk for CKD. The absolute risk, however, may vary by exposure to early, mid-, and late-life adversity.

Moreover, research on the relationship between PRS for kidney function and renal aging tends to draw on samples wholly composed of White adults, limiting the translation of these findings into clinical practices. Accordingly, research in this area must examine the interplay between social adversity throughout the lifecourse, PRS for kidney function, and renal aging among older Black and White adults. The findings gathered from this study can affect the field by clarifying the potential clinical value of PRS for kidney function.

There is also reason to believe that neighborhood contexts across the lifecourse may play a role in the development of CKD among older Black adults (23). Research suggests that neighborhood contexts characterized by impoverishment, racial segregation, perceived discrimination, psychosocial stressors, and other markers of disadvantage are predictors of adverse health outcomes and may be essential drivers in incidents and progress of CKD among adults (20,24,25). Research leads us to believe that Black children are more likely to reside in disadvantaged neighborhood contexts, which, in turn, may expose them to specific environmental stressors (e.g., crime and violence) that prompt stress and encourage unhealthy coping behaviors (26). Such contexts are also linked to deficiencies in the built environment, which directly affect behavioral risk factors. Research suggests that residing in disadvantaged neighborhood contexts gets “under the skin,” affecting health through wear and tear on the body associated with cumulative exposure adaptation to stressors (27). Taken together, the psychosocial factors listed above and contextual factors converge within neighborhoods to influence several psychosocial and genetic risk factors throughout the lifecourse, thus affecting both the development of CKD and how it relates to mortality in the lives of older Black adults. Moreover, neighborhoods are shaped by policies, not random occurrences. Accordingly, there is a need for policies focused on creating neighborhood-based programs that test for CKD, ensure behavioral intervention with program-planning approaches that explicitly consider cultural or ethnic factors, and make face-to-face interventions to ensure attendance at hemodialysis for individuals with ESRD (14,28).

The present study has several important strengths, including using a subsample of older Black participants from a large and representative sample of older adults. Our measure of CKD also derives from Cystatin C, a highly relevant marker of kidney function among older adults (29). Other research on kidney function among older adults has used a Cystatin C-based measure of kidney function and found links between this measure and health-related outcomes among older adults. Yet, it is essential to note that our study has limitations. First, the cross-sectional nature of the biomarker subsample linked to information on vital status among older adults limits our ability to examine all-cause mortality risk and its relation to kidney function change among older Black adults. As noted above, future work should investigate how gene and environmental conditions mediate and moderate the association between CKD and all-cause mortality risk among older Black adults.

The ongoing growth of older Black adults in the United States will bring significant numbers of individuals with CKD. Our results reveal that CKD was independently associated with a greater risk of death from all causes among a subsample of older Black adults from a survey of noninstitutionalized adults aged 50 and older in the United States. Our analysis finds that the relationship between CKD and all-cause mortality risk is independent of household income, years of education, and other known correlates of all-cause mortality among older Black adults. Most importantly, these factors do little to change the magnitude or significance of the association between CKD and all-cause mortality risk among older Black adults in the HRS.

## Data Availability

Our study uses secondary data that was not preregistered. The Health and Retirement Study is available to the public; please refer to its website for more information.
